# Single-cell RNA sequencing reveals cancer stem-like cells and dynamics in tumor microenvironment during cholangiocarcinoma progression

**DOI:** 10.3389/fcell.2023.1250215

**Published:** 2023-11-10

**Authors:** Jihye L. Golino, Jing Bian, Xin Wang, Jianyang Fu, Xiao Bin Zhu, Julie Yeo, Michael Kelly, Freddy E. Escorcia, Maggie Cam, Changqing Xie

**Affiliations:** ^1^ Thoracic and GI Malignancies Branch, Center for Cancer Research, National Cancer Institute, National Institutes of Health, Bethesda, MD, United States; ^2^ CCR Collaborative Bioinformatics Resource, National Cancer Institute, National Institutes of Health, Bethesda, MD, United States; ^3^ Frederick National Laboratory for Cancer Research, Leidos Biomedical Research, Inc., Frederick, MD, United States; ^4^ Molecular Imaging Branch, Radiation Oncology Branch, Center for Cancer Research, National Cancer Institute, National Institutes of Health, Bethesda, MD, United States; ^5^ NCI CCR Liver Cancer Program, Bethesda, MD, United States

**Keywords:** cholangiocarcinoma, cancer stem-like cells, tumoral heterogeneity, Tm4sf1, single-cell RNA sequence

## Abstract

Cholangiocarcinoma is a malignancy of the bile ducts that is driven by activities of cancer stem-like cells and characterized by a heterogeneous tumor microenvironment. To better understand the transcriptional profiles of cancer stem-like cells and dynamics in the tumor microenvironment during the progression of cholangiocarcinoma, we performed single-cell RNA analysis on cells collected from three different timepoints of tumorigenesis in a YAP/AKT mouse model. Bulk RNA sequencing data from TCGA (The Cancer Genome Atlas program) and ICGC cohorts were used to verify and support the finding. *In vitro* and *in vivo* experiments were performed to assess the stemness of cancer stem-like cells. We identified Tm4sf1high malignant cells as cancer stem-like cells. Across timepoints of cholangiocarcinoma formation in YAP/AKT mice, we found dynamic change in cancer stem-like cell/stromal/immune cell composition. Nevertheless, the dynamic interaction among cancer stem-like cells, immune cells, and stromal cells at different timepoints was elaborated. Collectively, these data serve as a useful resource for better understanding cancer stem-like cell and malignant cell heterogeneity, stromal cell remodeling, and immune cell reprogramming. It also sheds new light on transcriptomic dynamics during cholangiocarcinoma progression at single-cell resolution.

## 1 Introduction

Cholangiocarcinoma (CCA) is a lethal malignancy originating from the epithelial cells lining the bile ducts. Due to its rising global incidence (Bertuccio et al., 2019), heterogeneous pathology (Brindley et al., 2021), and resistance to conventional therapy (Brindley et al., 2021), there is growing need to characterize the CCA microenvironment to illuminate the molecular process and explore potential therapeutic strategies (Greten et al., 2023). Previous investigations have shown that CCA is highly desmoplastic and comprises entangled dense networks of inflammatory cells and the extracellular matrix (Brindley et al., 2021; Fabris et al., 2020; Zhang et al., 2020/11). However, dynamic change in cell composition and cell–cell crosstalk during CCA tumorigenesis has not been elaborated.

CSCs (cancer stem-like cells) are a rare subpopulation of tumor cells with strong tumorigenic capacity. These cells remain in a relatively quiescent state until exposed to various direct and indirect signals in the tumor microenvironment (TME). CSCs have been shown to play critical roles in tumor initiation, metastasis, chemotherapy resistance, and recurrence upon activation (Li et al., 2008; Wilson et al., 2011). Cancer stemness has been shown to be negatively associated with antitumoral immunity, suggesting that the presence of CSCs remodels the TME (Miranda et al., 2019; Galassi et al., 2021). This remodeling results in an immunosuppressive ecosystem partially through interactions between CSCs and surrounding stromal cells, which include immune cells (Miranda et al., 2019; Galassi et al., 2021). Historically, a broad spectrum of cell surface markers, including PROM1 and CD24, have been extensively used to identify CSCs, although the consensus has not been reached. Whereas tumor heterogeneity in CCA is a widely accepted phenomenon evidenced by scRNA-seq data, CSC heterogeneity in CCA has not been previously described.

The YAP/AKT CCA murine model is recognized as one of the most important preclinical models for hepatic stem-like CCA (Wang et al., 2018; Martin-Serrano et al., 2022). These mice develop tumors through a stepwise process (Zhang et al., 2017; Wang et al., 2018) and mimics the pathological procession of human CCA patients (Zhang et al., 2017), which validates the use of the YAP/AKT mouse model to study CCA *in vivo*. Moreover, this model serves as a valuable tool to study the characteristics of CSCs of CCA and their interactions with surrounding stromal/immune cells during tumorigenesis.

Here, we provide transcriptome analysis of 47,806 single cells, including hepatocytes, and epithelial and stromal cells from normal liver control and the other two different tumor progression timepoints of the YAP/AKT CCA mouse model.

## 2 Materials and methods

### 2.1 YAP/AKT CCA mouse model

In brief, a YAP/AKT CCA mouse model was established following the previous reports through hydrodynamically administered tail vein injections with 30 µg of YAP, 20 µg of AKT, and 2 µg of HSB2 plasmids (Li et al., 2015; Yamamoto et al., 2017; Zhang et al., 2021). The empty vectors with HSB2 were injected and used as controls for corresponding timepoints (e.g., Ctrl-W05 and Ctrl-W08). Normal liver samples were used as the baseline control (Ctrl, week 0). The presence of CCA and the extent of tissue infiltration were confirmed by an experienced murine histopathologist. All animals received humane care, and animal experiments were approved and conducted following the institutional guidelines and approved by the Animal Care and Use Committee of the NIH, Bethesda, Maryland, United States.

### 2.2 Library preparation and sequencing for mouse sample

Tissue samples were processed as single-cell suspensions (details are provided in Supplementary Methods S1). Single-cell sequencing was performed using the 10x Genomics Single Cell 3′v3.1 Reagent Kit according to the manufacturer’s instructions. Cell suspensions were assessed and counted by staining with acridine orange and propidium iodide fluorescence dyes on an automated cell counter (LunaFL, Logos Biosystems), and adjusted for single-cell partitioning to target approximately 6,000 datapoints per sample when possible. For single-cell library preparation, as defined in the 10x Genomics user guide, following cell partitioning with barcoded gel beads, the cells are lysed, and poly-adenylated transcripts are reverse-transcribed with the inclusion of a cell-specific barcode and a unique molecular identifier. Droplets are broken and barcoded cDNA is amplified for 14 cycles [amplification by a low number of cycles reduced the presence of heteroduplexes in the final PCR product (Thompson et al., 2002)] before Illumina-based sequencing libraries are prepared by fragmenting cDNA and adding necessary sequencing adapters along with a sample-specific index barcode. For sample preparation on the 10x Genomics platform, the Chromium Next GEM Single-Cell 3′ Kit v3.1 (PN-1000268), Chromium Next GEM Chip G Kit (PN-1000120), and Dual Index Kit TT Set A (PN-1000215) were used. The molarity of each library was calculated based on concentration and library size measured using a bioanalyzer (Agilent Technologies). Libraries were pooled and normalized to a final loading concentration. The sequencing run was setup as recommended with 28 cycles +10 cycles +10 cycles +90 cycles. Demultiplexing was performed using *cellranger* “mkfastq,” which allows for one mismatch in the sample index barcodes, and reads were aligned to a mm10 reference genome (refdata-gex-mm10-2020A) to generate a per-cell gene expression counts matrix with cellranger “count” (cellranger v6, 10x Genomics). A per-cell mean sequencing depth of 50,000 reads/cell was targeted for each sample. Libraries were sequenced on an Illumina NextSeq 2000 at the CCR Single-Cell Analysis Facility at NIH.

### 2.3 Cell lines and cell culture reagents

The human intrahepatic CCA cell lines HuCC-T1 and SNU-1079 were used for this study. Cells were cultured in RPMI1640-GlutaMAX™-I medium (Gibco, Grand Island, NY) supplemented with 10% FBS.

### 2.4 Tissue collection from the YAP/AKT CCA mouse model

All liver/tumor tissues were harvested at indicated timepoints, and the samples were minced into small pieces (*n* = 3/each timepoint). Sample pieces were processed using a tumor dissociation kit (Miltenyi Biotech # 130-095-929), following the manufacturer’s instructions. The cell suspension was subsequently filtered and counted to determine the concentration of live cells before being submitted for sequencing. In between, the samples were treated with Red Blood Cell Lysis Solution (Miltentyi # 130-094-183) and Debris Removal Solution (Miltenyi Biotec 130-109-398). The cells were maintained on ice during the isolation process.

### 2.5 Murine CCA scRNA-seq data analysis

Filtered feature-barcode matrix.h5 files from *cellranger* output for Ctrl, W05, and W08 samples were merged into a Seurat object through the Seurat workflow system (Satija et al., 2015). The sequencing data were preprocessed according to unique molecular identifier (UMI) counts, number of expressed genes, and mitochondrial content. Cells with low UMI counts (<500) or low complexity (<0.8 genes/UMI) were filtered from the subsequent analysis. Cells with gene or mitochondrial content exceeding three absolute deviations above the respective medians were filtered as well. The gene expression data were then normalized using the Seurat SCTransform function (Satija et al., 2015). Downstream analyses involving differential gene expression (DEG) and gene set variation analyses (GSVA) were performed within the NIH Integrated Analysis Portal (NIDAP) using R programs developed on the Palantir Foundry platform (Palantir Technologies). Analyses involving cell communication (CellChat and CellphoneDB), copy number variation (inferCNV), transcriptional factors and regulons (SCENIC), weighted gene correlation network (WGCNA), and stemness and pseudotime (CellTree and TSCAN) were performed on RStudio with custom code (will be submitted onto GitHub Pages). All scRNA-seq data were submitted to the Gene Expression Omnibus (GEO) public database at NCBI (GEO link pending). Publicly available scRNA-seq data from KRAS/p19 and YAP/AKT mouse models (GSE154170) (Affo et al., 2021) were used to verify the presence of Tm4sf1^high^ CSCs in other studies.

### 2.6 Determination of major cell types and their subpopulations

Highly variable genes were summarized by principal component analysis (PCA). The number of principal components used was determined using the elbow method, and the top 30 principal components were further projected (Becht et al., 2018). The Seurat FindClusters function was used for unsupervised clustering analysis (Satija et al., 2015). For each individual cell type, the average expression of previously published cell markers (Zhang et al., 2020/11) was calculated using the Seurat AddModuleScore function, and cells were classified based on the marker set with the highest average. Copy number variation (CNV) across epithelial cells was used to differentiate malignant cells determined using inferCNV (Chen et al., 2022/10), as we reported recently (Golino et al., 2023).

### 2.7 Differential expression analysis and gene set variation analysis (GSVA)

Differential gene expression analysis was performed on log-normalized data using the MAST algorithm (Finak et al., 2015) and through the Seurat “FindMarkers” function (Satija et al., 2015). Gene counts from malignant cells, cholangiocytes, and hepatocytes were aggregated prior to GSVA using the GSVA (version 1.47.0) R package. Pathways corresponding to significant gene sets were referenced from the C5:GO, CP:KEGG, and CP:REACTOME collections within the Molecular Signatures Database (MSigDB) (v2022.1.Mm). Pathways describing similar biological functions were omitted from visualization.

### 2.8 Cell identification

For each individual cell, the average expression of previously published stromal and immune cell markers (Zhang et al., 2020/11) was calculated using the Seurat AddModuleScore function. Cells were then classified according to the marker set with the highest average. Copy number variation (CNV) across epithelial cells was determined using inferCNV (Chen et al., 2022/10). Endothelial, fibroblast, and immune cells were pooled to form a reference dataset for inferCNV. A cutoff of 0.1 was used to filter cells having low gene counts, and an sd_amplifier value of 1.5 was used to filter background noise. A copy number score (CNS) was assigned for each cell through the following formula:

$$CNS=\Sigma \;{\left(CNV_{gene}-mean\left(CNV\right)\right)}^{2}.$$

Epithelial cells with a CNS in the top 25 percentile were further classified as malignant cells. The remaining epithelial cells were classified as cholangiocytes.

### 2.9 Cell differentiation analysis

The CellTree algorithm (version 3.16) (duVerle et al., 2016) was used to calculate a set of topic scores (measure of differentiation state) for each cell in the malignant, cholangiocyte, and hepatocyte populations. A set probability score was also calculated for each cell; this measure describes the likelihood of a cell having a particular topic score. The topic score with the highest probability was then assigned to each cell. Early-state malignant cells were labeled as those with topic scores below or equal to three. Early-state cholangiocytes and hepatocytes were labeled as those with topic scores below or equal to four. We also used CytoTRACE to define the differential or stemness status of human malignant cells from a previous study (Gulati et al., 2020). Cells were assigned a score to annotate differential/stemness status, with a higher score indicating higher stemness/characteristics.

### 2.10 Network analysis

We inferred gene regulatory networks using the SCENIC package (version 1.2.4) implemented in R (Aibar et al., 2017). The gene regulatory networks are inferred based on co-expression modules and TF motif enrichment analysis from scRNA-seq data. We calculated and ranked the activities of TFs, as measured by the regulon specificity score (RSS), using the AUCell package (version 3.12) (Suo et al., 2018). We also used the single-cell weighted gene co-expression network analysis (scWGCNA) R package (Langfelder and Horvath, 2008) to determine modules of co-expressed genes for malignant cell clusters. Clustering of genes was performed based on five k-nearest neighbors. A soft power of 18 was used to construct the co-expression network.

### 2.11 Pseudotime analysis

Pseudotime analysis was performed on malignant cell clusters using the Tools for Single-Cell ANaylsis (TSCAN) R package (version 1.32.0). Differentiation trajectory was drawn on Seurat PCA embeddings through modified exprmclust and plotmclust functions. An expression matrix containing the top 2,000 variable genes was used to infer genes controlling the direction of differentiation. The set of genes controlling the direction of differentiation was extracted through the TSCANorder and DIFFTEST functions. Genes having a q-value >0.05 were filtered from the list.

### 2.12 Cell–cell communication

Differential crosstalk and communication between stromal and immune cells across timepoints were analyzed using CellChat (version 1.1.3), as outlined by the procedure proposed by Jin et al. (2021). The default CellChat integrated database was used as the reference for inferring interaction signals. Cell communication groups containing fewer than 10 cells were filtered from the data. We used CellphoneDB (Efremova et al., 2020) to study the ligand–receptor interactions of early-state/late-state malignant cells with immune and stromal (endothelial and fibroblast) cells. Interaction pairs with a *p*-value <0.05 were considered significant.

### 2.13 Spheroid formation assay

The single-cell suspension of CCA cells was cultured in 6-well ultralow attachment plates (Corning Inc., New York, NY) at a density of 1,000 cells/well in a spheroid medium. The spheroid medium was prepared with DMEM/F12 medium supplemented with 1X B27 supplement (Gibco, Carlsbad, CA), human recombinant epidermal growth factor (hrEGF) (Gibco, Carlsbad, CA) (20 ng/mL), and bFGF (Gibco, Carlsbad, CA) (10 ng/mL). After incubating for 7 days, the number of spheroids was counted. The spheroid formation rate was assessed as the ratio of the number of spheroids/the number of cells cultured. Each group included triplicate wells.

### 2.14 Flow cytometry

Flow cytometry was used to detect cell populations with specific CSC surface markers. In brief, cultured cells were dissociated to single-cell suspension and washed with cold PBS two times before incubation with different antibodies for 30 min at 4°C. Cells were washed once more before performing flow cytometry analysis. The following antibodies were used for detecting stem cell markers by flow cytometry analysis: anti-CD24-PB human (BioLegend, San Diego, CA), anti-CD90-PE/Cy5 human (BioLegend, San Diego, CA), anti-CD133/2-APC human (Miltenyi Biotec, Milan, Italy), anti-LGR5-PE human (BioLegend, San Diego, CA), anti-CD44-PE/Cyanine7 human (BioLegend, San Diego, CA), anti-EpCAM-FITC human (Miltenyi Biotec, Milan, Italy), and anti-DCAMKL1-Alexa Fluor^®^ 488 (Abcam, Cambridge, MA). All abovementioned stained cells were then examined by using the CytoFLEX LX platform, and results were analyzed using FlowJo software version 10.4.2 (TreeStar Inc.).

### 2.15 RNA extraction and quantitative real-time (qRT)-PCR

Total RNA from cultured cells was extracted by using an RNeasy Mini Kit (Qiagen) according to the manufacturer’s instructions. cDNA was synthesized from 1 μg of total RNA by the Superscript III First-Strand Synthesis System (Invitrogen). cDNA samples were subjected to qRT-PCR amplification using custom primers on a Bio-Rad MyIQ detection system (Bio-Rad) according to the protocol provided by the manufacturer. Quantitative RT-PCR custom primers are described in Supplementary Table S6. GAPDH served as an internal standard.

### 2.16 Survival analysis and enrichment analysis

The survival analysis was performed on KMplot (http://kmplot.com/analysis/index.php?p=service&cancer=liver) (Nagy et al., 2018). We supplied multiple gene names to the KMplot by using the “use mean expression of selected genes” option. The enrichment analysis was performed on MsigDB (https://www.gsea-msigdb.org/gsea/msigdb) with default parameters (Liberzon et al., 2011). The gene expression profile of liver cancer patients was downloaded from the TCGA (https://portal.gdc.cancer.gov/) and ICGC (https://dcc.icgc.org/) websites. Raw read counts were extracted from files with the suffix “htseq.counts.” The trimmed mean of M-values (TMM) normalized expression value was generated by the “edgeR” package (Robinson et al., 2010). The clinical information was also downloaded from the TCGA and ICGC websites.

### 2.17 Statistics analysis

All statistical analyses were performed using R (version 4.1.0, http://www.rproject.org) or GraphPad Prism (version 7.04). The differences between groups were analyzed by the chi-squared test, Student’s *t*-test, Wilcoxon test, Pearson correlation test, and Kruskal–Wallis test, when appropriate. All tests were two-sided, and a *p*-value < 0.05 was considered statistically significant.

## 3 Results

### 3.1 Single-cell transcriptome characterizes heterogeneous malignant cells in YAP/AKT mouse CCA

**FIGURE 1:**
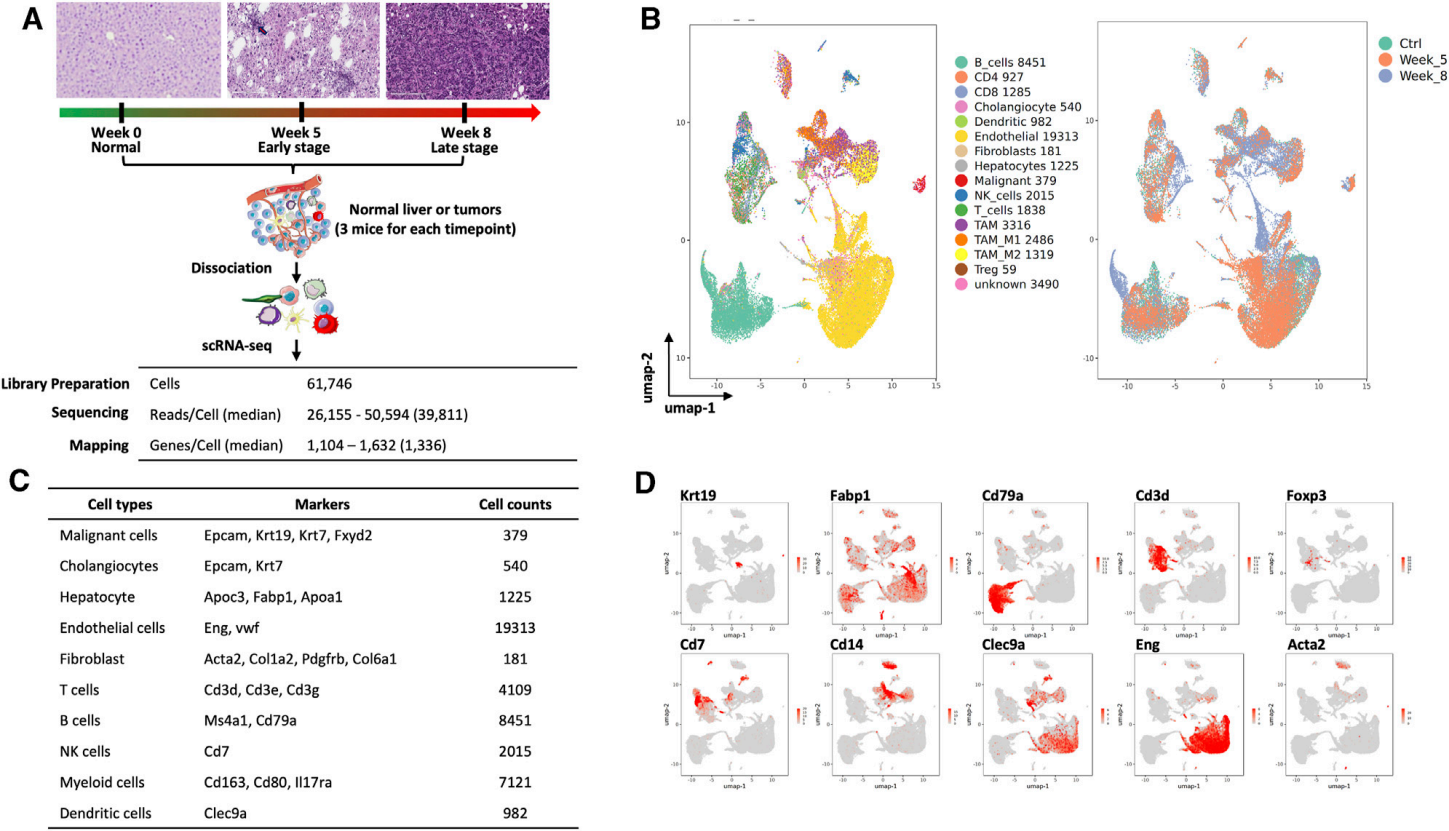
Single-cell analysis of liver and cholangiocarcinoma from YAP/AKT mice. **(A)** A schematic diagram highlighting the workflow including isolation and sequencing of single cells for this study. Single cells were prepared from the liver tissues/tumors of YAP/AKT CCA mice at control (baseline) and different tumor progression timepoints, including week 5 (W05) and week 8 (W08). The transcriptome of single cells was sequenced using the 10x Chromium system. **(B)** The UMAP plot of 47,806 single cells to visualize cell-type clusters based on the expression of known markers (left panel) and isolated cells at indicated tumor progression stages, including control, week 5, and week 8. **(C)** Cell counts and markers used to annotate the known cell types, including epithelial cells, hepatocytes, immune cells, endothelial cells, and fibroblasts, from the liver tissue/tumors from YAP/AKT CCA mice. **(D)** The individual gene UMAP plots showing the expression levels and distribution of representative markers of known cell types, which distinctly separates epithelial cells, hepatocytes, immune cells, endothelial cells, and fibroblasts from the liver tissue/tumors from YAP/AKT CCA mice.

**FIGURE 2:**
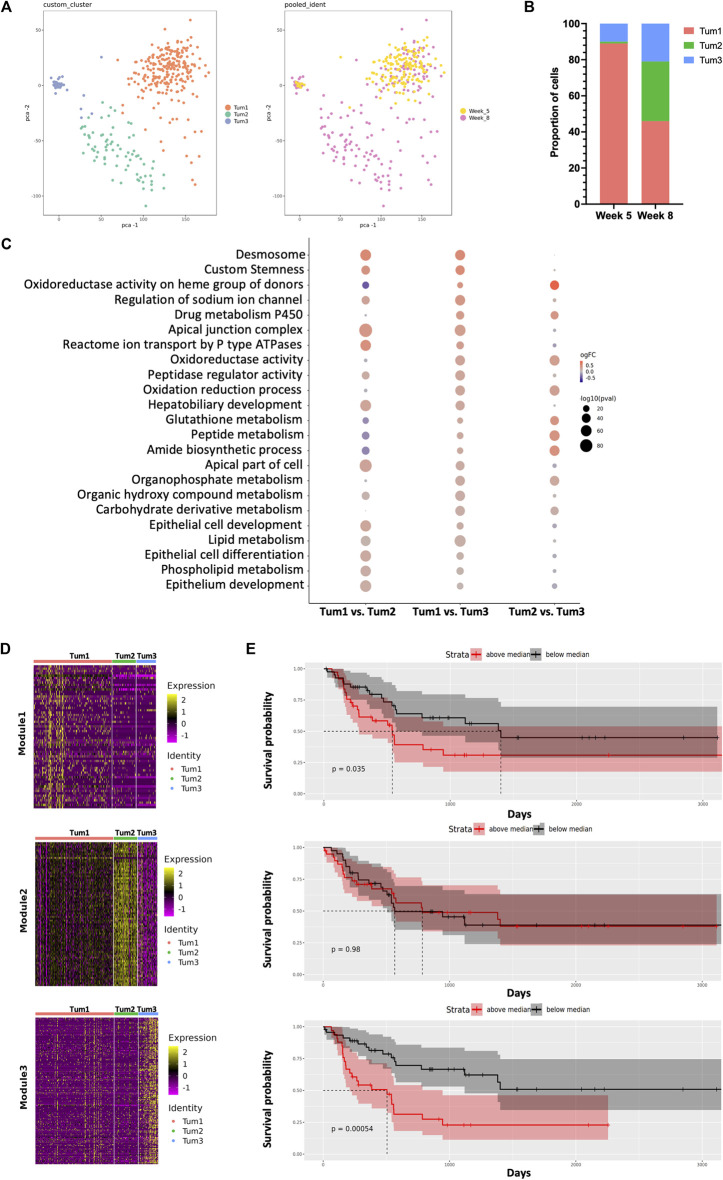
Dynamic intratumoral malignant cell heterogeneity in the CCA of YAP/AKT mice. **(A)** The PCA plot of 379 malignant epithelial cells from YAP/AKT mice colored by cluster (left panel) and timepoint (right panel). **(B)** The proportion of malignant cell subclusters in different timepoints of tumor progression. **(C)** Function enrichment analysis for differentially expressed genes between subclusters of malignant cells in **(A)**. **(D)** Heatmap of expression profile of signature genes in indicated modules identified by WGCNA (weighted gene co-expression network analysis). A summary list of genes associated with the corresponding WGCNA module is shown in Supplementary Table S2. **(E)** The corresponding Kaplan–Meier overall survival curves of patients were grouped by the gene signatures in WGCNA modules **(D)** based on the ICGC cohort.

We obtained single-cell transcriptomics from normal control (week 0, Ctrl), W05 and Ctrl-W05, and W08 and Ctrl-W08 livers or tumors of the YAP/AKT mouse model (Figure 1A). H&E staining from W05 (week 5) samples showed atypical epithelial/ductular proliferation, indicating the initiation stage of cancer, whereas W08 (week 8) samples showed carcinoma formation (Figure 1A, Supplementary Figure S1). Therefore, W05 samples were denotated as being in the early stage of CCA and W08 samples were denotated as being in the late stage of CCA. Overall transcriptomic patterns overlapped among the samples from normal livers (Ctrl), Ctrl-W05, and Ctrl-W08, indicated in the PCA plot (Supplementary Figure S2). Therefore, a total of 47,806 single cells were extracted from murine livers or tumors derived from Ctrl, W05, and W08 samples for subsequent analysis. A total of 16 distinct cell clusters were identified (Figures 1B–D). The cells comprised mainly of clusters of endothelial cells, hepatocytes, epithelial cells, immune cells, and fibroblasts based on the expression of known markers (Figure 1D, Supplementary Figure S3). As expected, the epithelial cells were composed of malignant cells and cholangiocytes, which is consistent with the cellular characteristics of the liver (Zhang et al., 2020/11; Massalha et al., 2020; Ma et al., 2019). The immune cells comprised CD4 cells, CD8 cells, regulatory T cells (Tregs), NK cells, B cells, and myeloid cells, which included dendritic cells, TAM1 and TAM2 (Figures 1B–D). Next, we further analyzed epithelial cells, especially malignant cells, from the epithelial cell cluster. The malignant epithelial cells were clustered into three separate subsets (Tum1, Tum2, and Tum3) (Figure 2A). Notably, the proportion of the subset of Tum2 expanded in the late carcinoma of W08 (Figure 2B).

In terms of malignant cell composition, Tum1, Tum2, and Tum3 contained 89%, 1%, and 10% of W05 cells, respectively, and 46%, 33%, and 21% of W08 cells, respectively. The clusters were distinctively separate from each other, characterized by a unique transcriptional profile of cells. Functional enrichment analysis revealed that differentially expressed genes (Supplementary Figure S4, Supplementary Table S1) across the three subclusters corresponded to various important pathways. For instance, Tum1 cells were enriched with pathways associated with epithelial cell development and differentiation, ion transmembrane transport, peptidase regulator activity, and lipid metabolic processes. Tum2 cells were enriched with pathways concerning the metabolic processing of glutathione, peptide, and amide and oxidation reduction process, whereas Tum3 cells were enriched with pathways associated with the phospholipid metabolism (Figure 2C).

To gain a more detailed view of the changes in gene expression patterns among the subset of malignant cells, we performed weighted gene correlation network analysis (WCGNA) (Langfelder and Horvath, 2008) on the malignant cell clusters. We identified panels of specific signatures correlating with the subsets of malignant cells during CCA progression. The expression of the genes in module 1 gradually decreased from left to right, whereas the expression of genes in module 3 increased from left to right (Figure 2D, Supplementary Table S2). There was a significant relationship between the gene matrix of these two modules and the clinical outcome of CCA patients (ICGC cohort). Notably, patients with either of two of these highly expressed gene signatures (modules 1 and 3) showed worse prognosis, suggesting potential predictive values of these gene signatures (Figure 2E). Among these genes, we identified several factors that were related to tumor progression and were found to be consistent with previously reported, for example, CAT (Loilome et al., 2012), ATP1B1 (Singhi et al., 2020), CCND1 (Zhao et al., 2008), and CXCL12 (Miyata et al., 2019) (Supplementary Figure S5). By utilizing the l2p R package, we can map genes in the individual module to key functional pathways, notably cellular differentiation and development in module 1, whereas genes in the Tum3 module play pivotal roles in VEGF-mediated vascular endothelial growth, which is evidenced by the presence of genes such as FLT1, FLT4, KDR, NOTCH1, NRP2, NRP1, and ENG. Genes within the Tum2 module are predominantly ribosomal in nature and align closely with pathways associated with protein translation.

### 3.2 Single-cell transcriptome characterizes stemness and heterogeneity of CSCs in YAP/AKT mouse CCA

**FIGURE 3:**
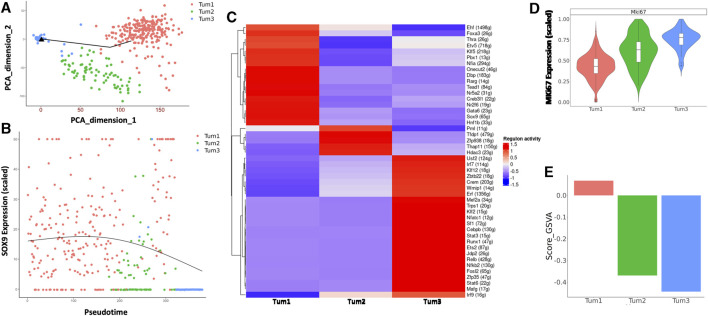
Characterization of the CSC cluster in CCA malignant cells of YAP/AKT mice. **(A)** Pseudotime trajectory analysis of the three subclusters of malignant cells annotated by subclusters of malignant cells. **(B)** The trend of Sox9 expression along the pseudotime trajectory of the three subclusters of malignant cells. **(C)** A heatmap of regulon scores from the SCENIC (single-cell regulatory network inference and clustering) analysis. Rows, individual regulons. Columns, cells organized according to re-clustering of malignant cells. **(D)** Violin-plot showing the relative MKi67 expression between subclusters of malignant cells. **(E)** Bar-plot showing the average GSVA stemness score for each malignant cell cluster.

Next, we focused on the dynamic changes that occurred within malignant cells during the progression of CCA. To this end, we performed pseudotime analysis on malignant cells obtained from weeks 5 and 8 (Figure 3A). The genes used to construct the pseudotime trajectory are listed in Supplementary Table S3. There is a correlation between cluster membership and pseudotime state, with most cells from Tum1 being positioned at the start of the trajectory, whereas cells from Tum3 were positioned at the end of the trajectory. Moreover, cells from Tum2 were located between the start and end points of the trajectory plot (Figure 3A), suggesting that these cells were an intermediary population of malignant cells during CCA progression. The expression of Sox9, which is one of the critical TFs that control the fate of CSCs, also decreases toward the later pseudotime states (Figure 3B). These results suggest a transition in malignant cell differentiation over time, as indicated by the trend where more W08 cells were found in Tum2 and Tum3 than in Tum1 (Figure 2B).

We used SCENIC (Yamamoto et al., 2017) to investigate the expression of transcriptional regulons across the different malignant cell subclusters. Among transcriptional regulators, we found a significant number of high-confidence regulons that were distinctively expressed across all three subclusters, such as Foxa3, Klf5, Tead1, Gata6, Sox9 in Tum1; Pml and Hdac3 in Tum2; and Klf12, Cebpb, Stat3, Nfkb2, and Irf9 in Tum3. These distinct regulon sets suggest a potential role in determining malignant cell heterogeneity (Figure 3C). Furthermore, the proliferation activity was different across the three malignant cell subclusters, with Tum1 having the lowest MKi67 expression and Tum3 having the highest Mki67 expression (Figure 3D). We used a list of previously published CSC-related genes (Supplementary Table S4), such as Sox9, Aldh3a2, Cd24a, Prom1, and Cd44a, as a spike-in for GSVA. We calculated the enrichment score of cancer stemness in each malignant cell cluster using the GSVA and found a significantly higher stemness signature in Tum1 than in the other clusters (Figure 3E, Supplementary Figure S6A), indicating that Tum1 is a group of malignant cells with stem-like features.

CCA heterogeneity has been well illuminated (Ho et al., 2019; Ma et al., 2019), but the heterogeneity of CCA CSCs remains unclear. To reveal the heterogeneity of CSCs, stem-like cell cluster Tum1 was re-clustered into three subclusters, indicating the heterogeneity of CSCs (Supplementary Figure S6B). We explored the potential regulation mechanism with SCENIC, which showed distinguished transcriptional regulons of these three subclusters of CSCs, suggesting that these regulons may be drivers of the heterogeneity of CSCs (Supplementary Figure S6C).

In summary, our data revealed both the similarities and dissimilarities in gene expression patterns among different malignant cell clusters. We also defined the Tum1 cell population with the features associated with CSCs of CCA, including characteristics of relative quiescence and a high stemness score with specific TF expression patterns. Nevertheless, our data suggest heterogeneity of the CSC population.

### 3.3 Tm4sf1+ malignant cells are highly tumorigenic cells during CCA development

**FIGURE 4:**
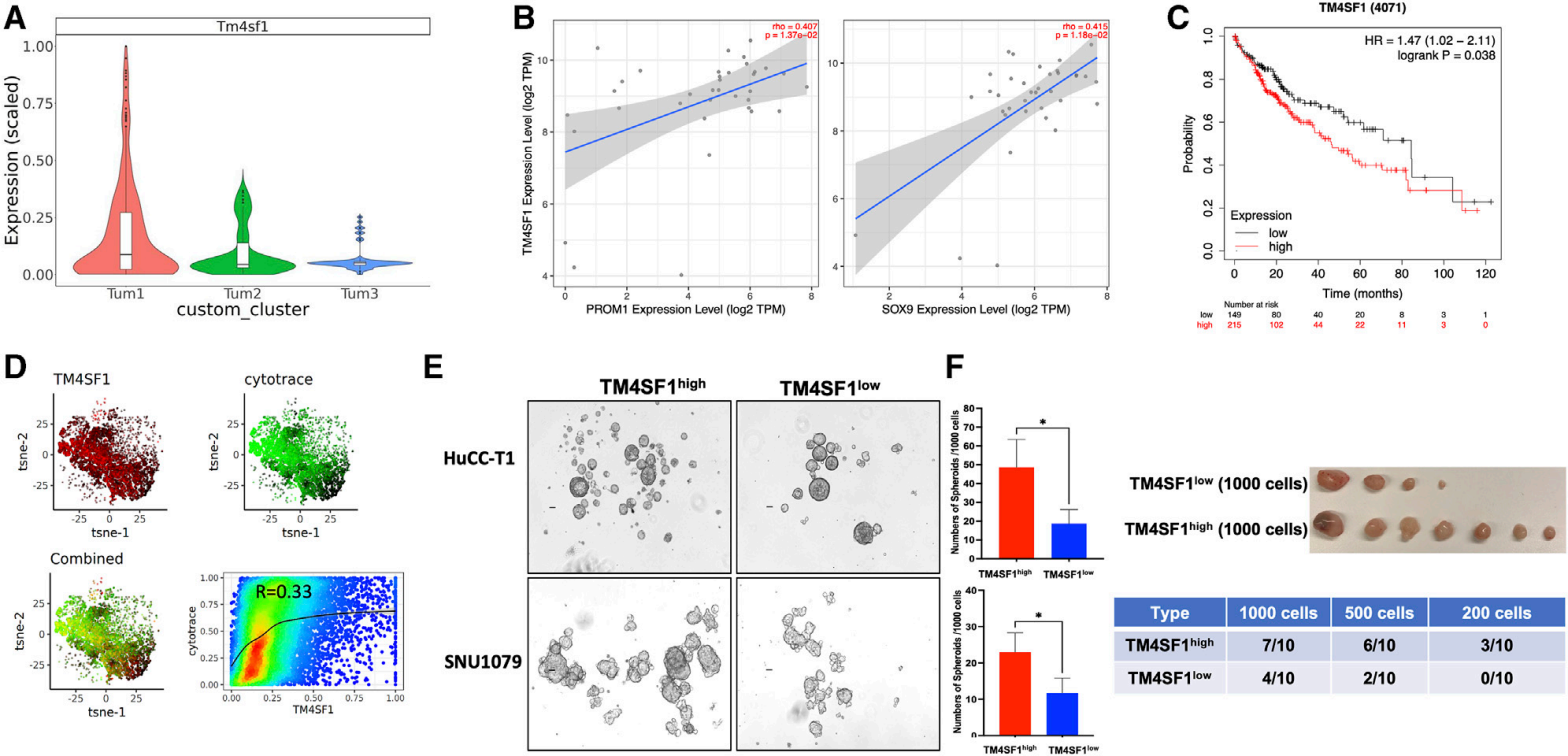
TM4SF1 is a potential marker for the CSC cluster in CCA malignant cells. **(A)** Bar-plot showing the relative Tm4sf1 expression level between subclusters of malignant cells. **(B)** Association of TM4SF1 expression with the expression of CSC markers PROM1 (left panel) and SOX9 (right panel) in the CCA cohort. Scatterplots were generated using the Tumor IMmune Estimation Resource (TIMER) web tool (https://cistrome.shinyapps.io/timer/) to identify the expressions of PROM1 and SOX9 that are associated with TM4SF1 expression in the CCA cohort of the TCGA database. **(C)** Kaplan–Meier survival curves of the overall survival of the liver cancer cohort from the Human Protein Atlas datasets for TM4SF1 gene expression stratified by high (red) or low (green) expression levels. **(D)** tSNE showed TM4SF1 expression of human iCCA scRNA-seq data (GSE138709) and correlation with CytoTRACE score (R = 0.33). **(E)** Representative images of spheroids from HuCC-T1 and SNU1079 CCA cell lines. The bar graph shows the spheroid-forming capacity of TM4SF1+ cells determined by tumor spheroid assays (1,000 cells/well). *p < 0.05. The cell suspension was cultured in DMEM/F12 medium supplemented with 1X B27 supplement, hrEGF (20 ng/mL), and bFGF (10 ng/mL) for 7 days. The number of spheroids was counted. **(F)** Tumor-initiating capacity test from TM4SF1high HuCC-T1 cells. The upper panel shows tumor image from 1 × 10^3^ TM4SF1^high^ and TM4SF1^low^ cells. The lower table shows the tumor formation numbers from three different diluted cell numbers.

As stated earlier, cluster Tum1 was a group of malignant cells with stem-like features. Among the differentially expressed genes between Tum1 and Tum2/3, Tm4sf1 is a cell membrane protein that was recently considered as a new marker of CSCs in breast cancer, lung cancer, and melanoma (Hong et al., 2022), and is expressed relatively higher in Tum1 (Figure 4A, Supplementary Table S1). Using the other two publicly available scRNA-seq datasets, including one mouse sample from the YAP/AKT CCA mouse model and the other mouse from the KRAS/p19 CCA model, we found similar three clusters, with one of them having the highest stemness score and a relatively higher expression of Tm4sf1 (Supplementary Figure S7). Correspondingly, we found that the expression of the TM4SF1 gene is positively correlated with well-known CSC markers PROM1 and SOX9 based on bulk RNA sequence data from the TCGA human CCA cohort (Figure 4B). Interestingly, patients with a high expression of TM4SF1 in liver tumor samples had worse overall survival (Figure 4C). In addition, scRNA-seq analysis of human iCCA samples confirmed this positive correlation of TM4SF1 expression with stemness scores assigned by the CytoTRACE (Becht et al., 2018; Affo et al., 2021) (Cellular (Cyto) Trajectory Reconstruction Analysis using gene Counts and Expression) score (Figure 4D). These findings demonstrate the strong correlation among TM4SF1 expression, cancer stemness, and clinical outcome. Moreover, TM4SF1 expression levels and positivity were different between two human CCA cell lines (Supplementary Figure S8A). Importantly, CSC markers CD24, PROM1, and SOX9 were much higher in TM4SF1^high^ cells of CCA cell lines, including HuCC-T1 and SNU1079 cells, than in TM4SF1^low^ cells (Supplementary Figure S8B). TM4SF1^high^ CCA cells formed more tumor spheroids than TM4SF1^low^ cells (Figure 4E). Nevertheless, the serial limiting dilution transplantation assay showed that TM4SF1^high^ generated significantly more tumors in nude mice (Figure 4F) and had a shorter latency period than TM4SF1^low^ cells. Taken together, the abovementioned data suggest that TM4SF1 could be a new cell membrane marker of CSCs of CCA. These analyses underline the clinical significance of TM4SF1^high^ cells for CCA, indicating that TM4SF1^high^ cells may play a critical role in CCA tumorigenesis.

### 3.4 Dynamic changes of stromal cells and interaction in high/low stemness malignant cells during tumor progression

**FIGURE 5:**
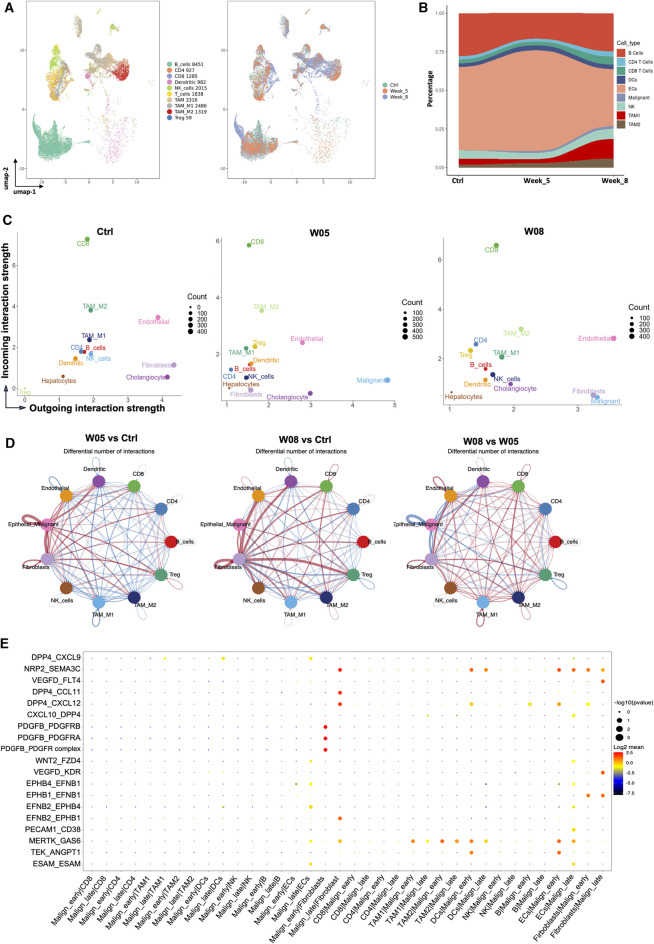
Dynamic change of interaction of cancer stem-like cells with stromal cells during CCA development. **(A)** UMAP plot of immune cells was grouped into 10 cell subtypes and indicated by color (left panel) and timepoints (right panel). **(B)** Sankey plot showing dynamic changes in the proportion of stromal cells along with tumor progression in YAP/AKT CCA mice. **(C)** Scatterplot of incoming and outgoing interaction strengths of each cell population at the baseline (Ctrl) and change of strength between control and different timepoints (W05 and W08). Ctrl, control; W05, week 5; W08, week 8. **(D)** Circle plot displaying putative ligand–receptor interaction between different cell types, with the width of edges representing the numbers of the communication. The edge colors are consistent with the signal sender. A thicker edge line indicates a stronger signal. **(E)** Bubble plot of the communication probability of all the significant ligand–receptor pairs that contributed to interaction between malignant cells and various stromal cells. The dot color and size represent the communication probability and *p*-values. *p*-values were computed from the one-sided permutation test.

To explore the dynamic changes of the stromal cells during tumor progression, we first evaluate the dynamic changes of stromal cells that occurred during tumor progression in YAP/AKT CCA mice. By re-clustering the stromal cells, we obtained 10 cell clusters (Figure 1B and Figure 5A), which comprised T cells, B cells, myeloid cells, fibroblasts, and endothelial cells. There was a dynamic change in the proportion of stromal cell populations as the tumor progressed (Figure 5B). Among immune cells, the proportion of B cells decreased at W05 but remained constant by W08, which may reflect a response to plasmid exposure rather than changes due to tumor progression. The proportion of pro-tumoral TAM2 gradually increased with tumor progression, whereas the proportion of antitumoral TAM1 remained at similar levels from W0 to W05 but dramatically increased by W08 (Figure 5B). We used Cellchat (version 1.1.3)^29^ to further study the interactions between cell populations during CCA progression in YAP/AKT mice. Through signal strength plots and cellular communication networks, we noticed a shift in the crosstalk across timepoints. At the baseline, endothelial cells, fibroblasts, and cholangiocytes have the strongest outgoing interaction signals (Figure 5C), such as Kit and Pecam from endothelial cells and Spp1 and Alcam from cholangiocytes (Supplementary Figure S9A). At W05, the outgoing signals from fibroblasts and cholangiocytes became attenuated, whereas signals from malignant cells became the strongest. Finally, at W08, there was a rebound in fibroblast outgoing signaling. This shift from cholangiocytes to malignant cell crosstalk appears to be toward fibroblast crosstalk and increases not only in strength but also in the number of interactions (Figure 5D). Interestingly, throughout all three timepoints, CD8 T cells had the persistently strongest incoming interaction signal (Figure 5C, Supplementary Figure S9B). Overall, the number and intensity of cell–cell interactions in the W08 CCA samples were higher than those in the W05 CCA samples (Figure 5D), suggesting evolving complexity of the interaction during tumor progression. With CellPhoneDB analysis, we found that the high stemness malignant cell cluster (malignant-early) had differential interactions with stromal cells in comparison to that of the low stemness malignant cell cluster (malignant-late) during the CCA progression (Figure 5E). For example, there were strong interactions between the high stemness malignant cell population and fibroblasts through the ligand:receptor interaction of PDGFb:PDGFR. However, this interaction was less prominent for the low stemness malignant population and replaced with DPP4:CCL11 and DPP4:CXCL12 pairings. Although the interaction between T cells and high/low stemness malignant cells did not significantly change, we noted differences of the interaction between myeloid cells and high/low stemness malignant cells. We also observed differential interactions between the endothelial cells and high stemness malignant cells versus low stemness malignant cells, such as TEK:ANGPT1 versus MERTK:GAS6, respectively.

## 4 Discussion

To our knowledge, this is the first study to define the Tm4sf1^high^ malignant cells as CSCs in CCA. We also presented the data showing dynamic changes in cell composition of different cells and dynamic interaction among CSCs, stromal cells, and immune cells during CCA tumorigenesis in the YAP/AKT mouse model.

The traditional method to identify CSCs from CCA focuses on sorting by FACS based on cell-surface markers, but derived CSCs are usually a mixture of CSC populations (McGrath et al., 2020). Using the YAP/AKT CCA mouse model and high-resolution scRNA-seq, we revealed that malignant cells display transcriptomic heterogeneity. Functional analysis demonstrated that malignant cell subclusters were enriched with unique functions, including those related to metabolic processes, stemness, and immune response, which may reflect the functional heterogeneity among malignant cells during tumor progression. We identified a subset of malignant cells with stemness features and metabolic functions along tumor progression. These functions were uniquely enriched in this subpopulation and reflect distinct requirements for CSCs. It will be helpful to gain further insight regarding functional requirements for stemness maintenance of CSCs in CCA. In this study, we demonstrated that TM4SF1 is a novel cell surface marker of CSCs in CCA, which may be one of the potential targets for CCA treatment. Although we used a similar YAP/AKT model as previously reported (Affo et al., 2021), the data generated different impressions in terms of Tm4sf1 in high stemness cell population, which may be explained by the different sampling timing and scRNA-seq process. Indeed, efforts have been taken to develop agents to block TM4SF1 (Chen et al., 2022/10; Visintin et al., 2015). Our data indicated the proportion of CSCs with Tm4sf1^high^ reduced at week 8 in comparison to week 5. It is unclear whether CSCs are enriched gradually from the time of tumor initiation to the stage of tumor metastasis or along with tumor size/number increases in CCA, which needs to be explored with increasing timepoints of this specific CCA model and other models.

Previous evidence has showed that CSCs contribute to the education and reconstitution of the immune microenvironment in CCA (Raggi et al., 2017; Zeng et al., 2018). Conversely, others have demonstrated that the immune microenvironment promotes the generation and maintenance of CSCs (Raggi et al., 2015). However, this bi-directional communication is dynamically changed. Here, we revealed these dynamic changes within CSC populations, as well as the interaction with surrounding stromal cells. For example, the CXCL12–DPP4 interaction between fibroblasts and tumors and the role of the PDGFR pathway on CCA tumorigenesis have been elaborated (Affo et al., 2021). Our data suggest that this interaction is dynamic rather than static. In addition, the proportion of immune cell types T, B, NK cells, and macrophages changed as the tumor progressed, which likely suggests that these cells aid in the survival of CSCs and propagate tumorigenesis.

We noticed a low yield of the malignant cell population in our study, which may be related to the cell loading limitation of the scRNAseq platform without specific enrichment for epithelial cells. Our findings provide insight into the cellular heterogeneity of CCA at the different developmental timepoints, which facilitates a deeper understanding of CCA pathogenesis. Furthermore, our findings on the crosstalk between CSCs and stromal cells provide potential strategies for the exploration of combining immunotherapy and CSC-targeted therapies for precision medicine. Confirmatory *in vitro* and *in vivo* experiments will help elucidate the underlying biological mechanisms and are ongoing.

In conclusion, our scRNA-seq analysis of tumor progression over time in the YAP/AKT CCA mouse model identified Tm4sf1^high^ malignant cells as potential CSCs and observed dynamic TME during CCA tumorigenesis. These findings provide important insights into CCA tumorigenesis mechanisms, which can aid in the development of effective therapeutic strategies with immunotherapy and beyond.

## Data Availability

The datasets presented in this study can be found in online repositories. The names of the repository/repositories and accession number(s) can be found at https://www.ncbi.nlm.nih.gov/geo/, GSE233623.

## Abbreviations

CCA, cholangiocarcinoma; CSC, cancer stem-like cells; CytoTRACE, cellular (Cyto) trajectory reconstruction analysis using gene counts and expression; DEGs, differential expressed genes; GEO, Gene expression omnibus; GSVA, gene set variation analyses; ICGC, international cancer genome consortium; PCA, principal component analysis; SCENIC, single-cell regulatory network inference and clustering; scRNAseq, single-cell RNA sequencing; TCGA, The Cancer Genome Atlas program; TIMER, tumor immune estimation resource; TME, tumor microenvironment; Treg, regulatory T cells; t-SNE, t-distributed stochastic neighbor embedding; UMAP, uniform manifold approximation and projection; WGCNA, weighted gene co-expression network analysis.
