# Supplementary material for: Single-cell RNA sequencing reveals cancer stem-like cells and dynamics in tumor microenvironment during cholangiocarcinoma progression
Source: Front Cell Dev Biol. 2023 Nov 10;11:1250215. doi: 10.3389/fcell.2023.1250215 (PMC10667919; doi:10.3389/fcell.2023.1250215)
Supplement: Supplementary file 3 [file Presentation1.pdf]

Supplementary Material

1 Supplementary Figures

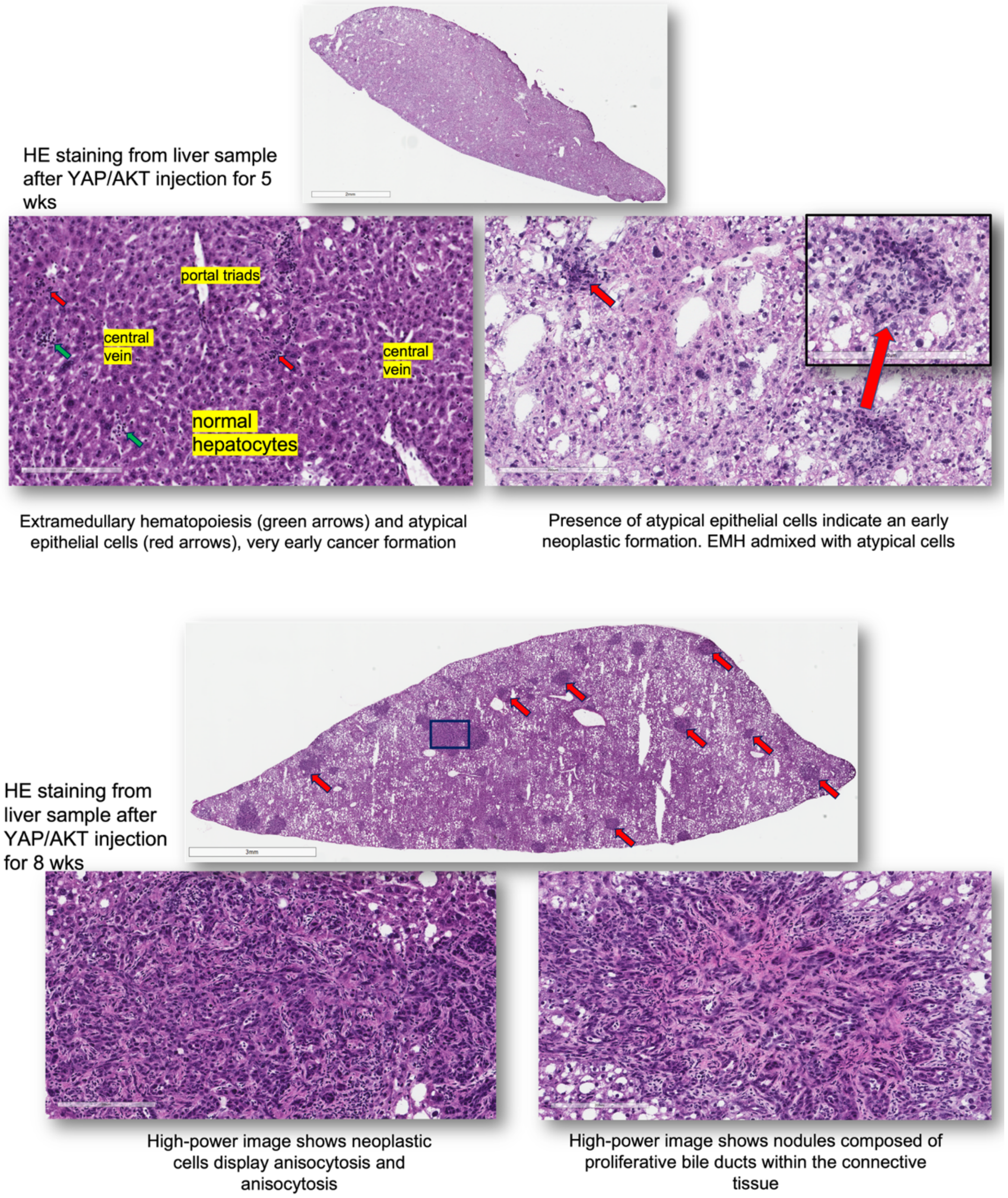

Supplemental Figure 1. Representative H&E staining from liver samples from Week 5 (left panel) and Week 8 (right panel) after YAP/AKT plasmid injection through the tail vein.

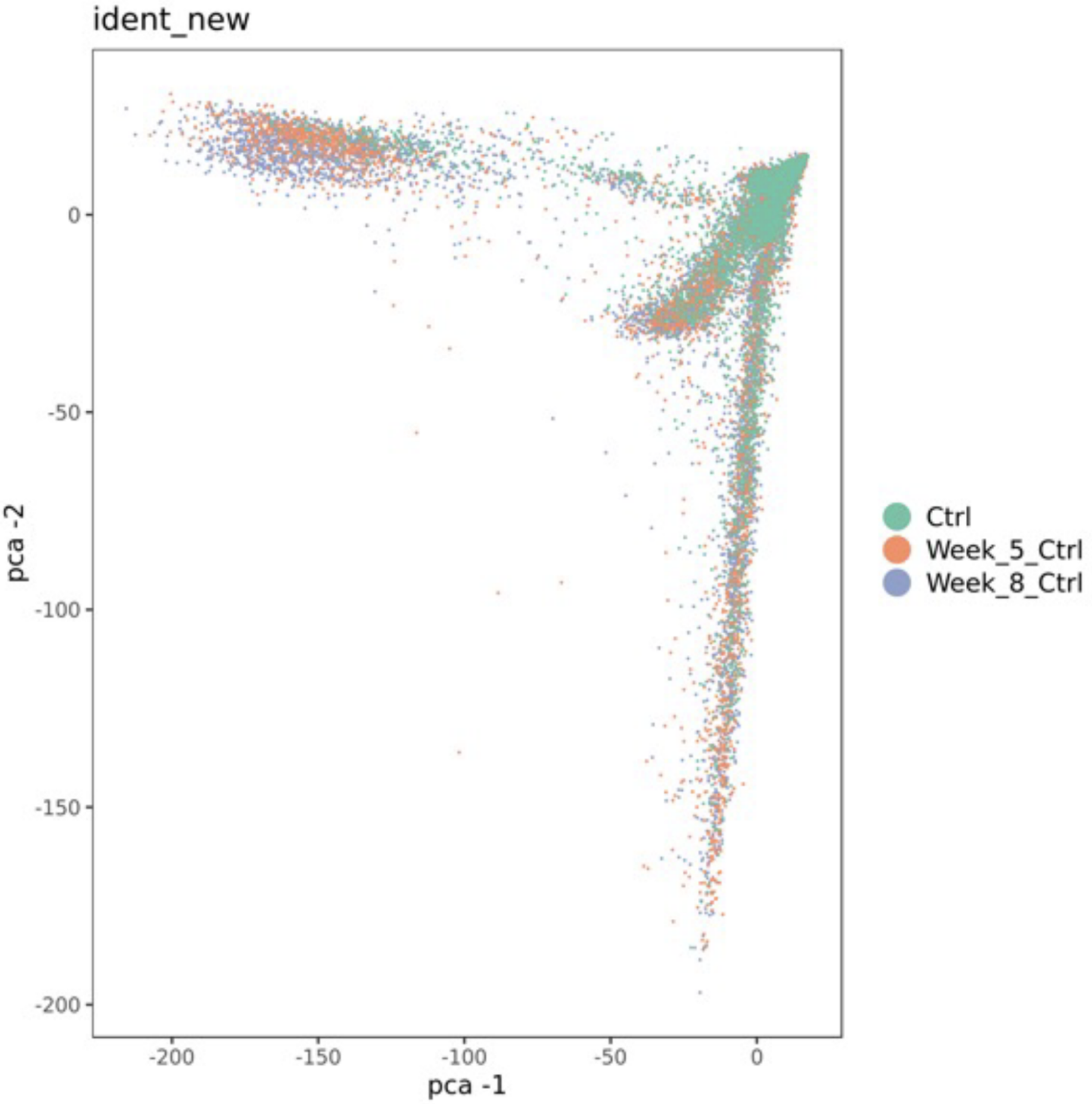

Supplemental Figure 2. PCA plot of transcriptomics derived from normal livers (Ctrl), and liver samples from Ctrl-W05 and Ctrl-W08 (empty plasmid injection).

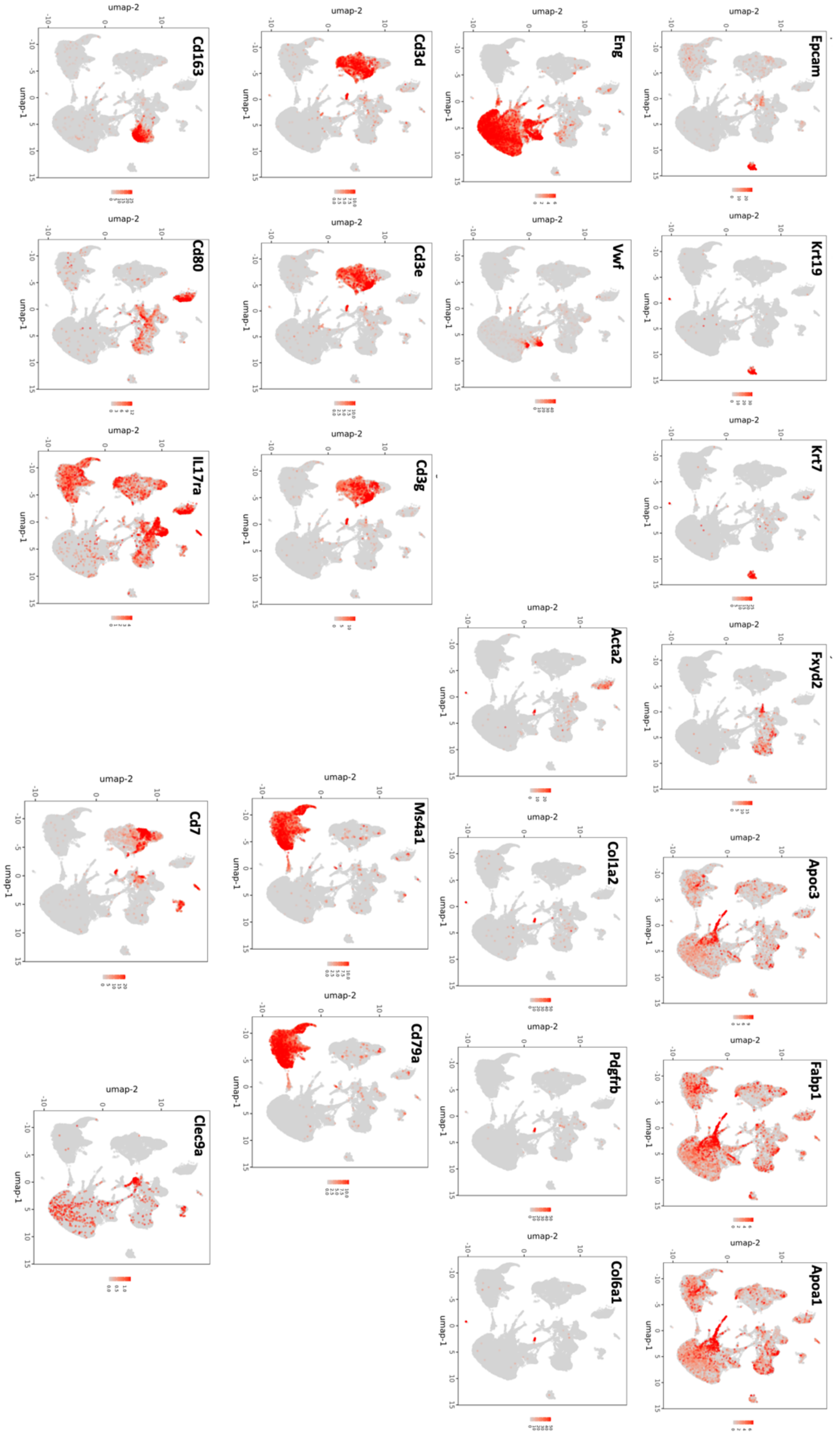

Supplemental Figure 3. The UMAP plots showing the expression levels and distribution of representative canonical markers of known cell types, which distinctly separates epithelial cells, hepatocytes, immune cells, endothelial cells and fibroblasts from the liver tissue/tumors from YAP/AKT CCA mice.

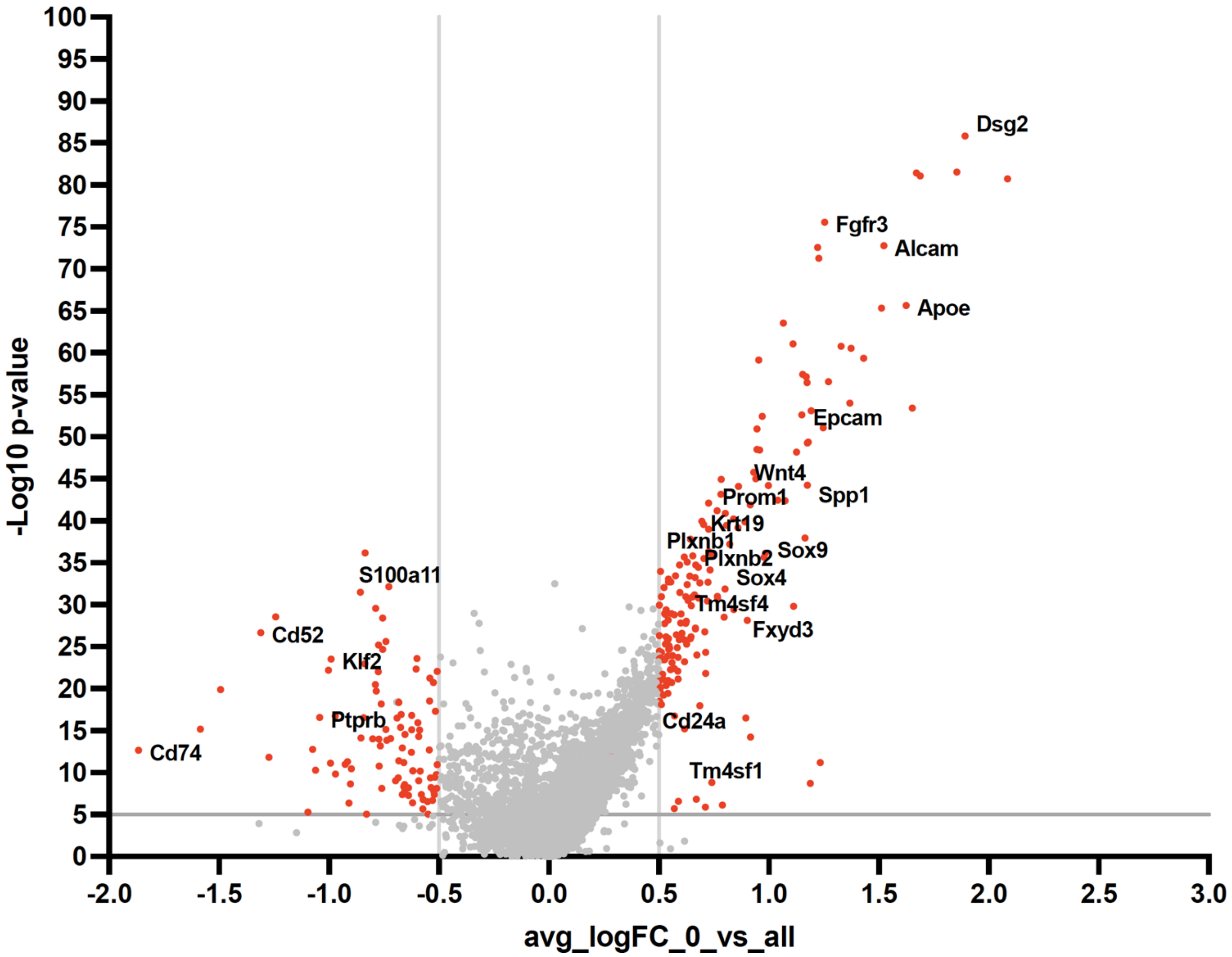

Supplemental Figure 4. Volcano plot showed differentially expressed genes between Tum1 malignant cell and other malignant cells from YAP/AKT CCA mice.

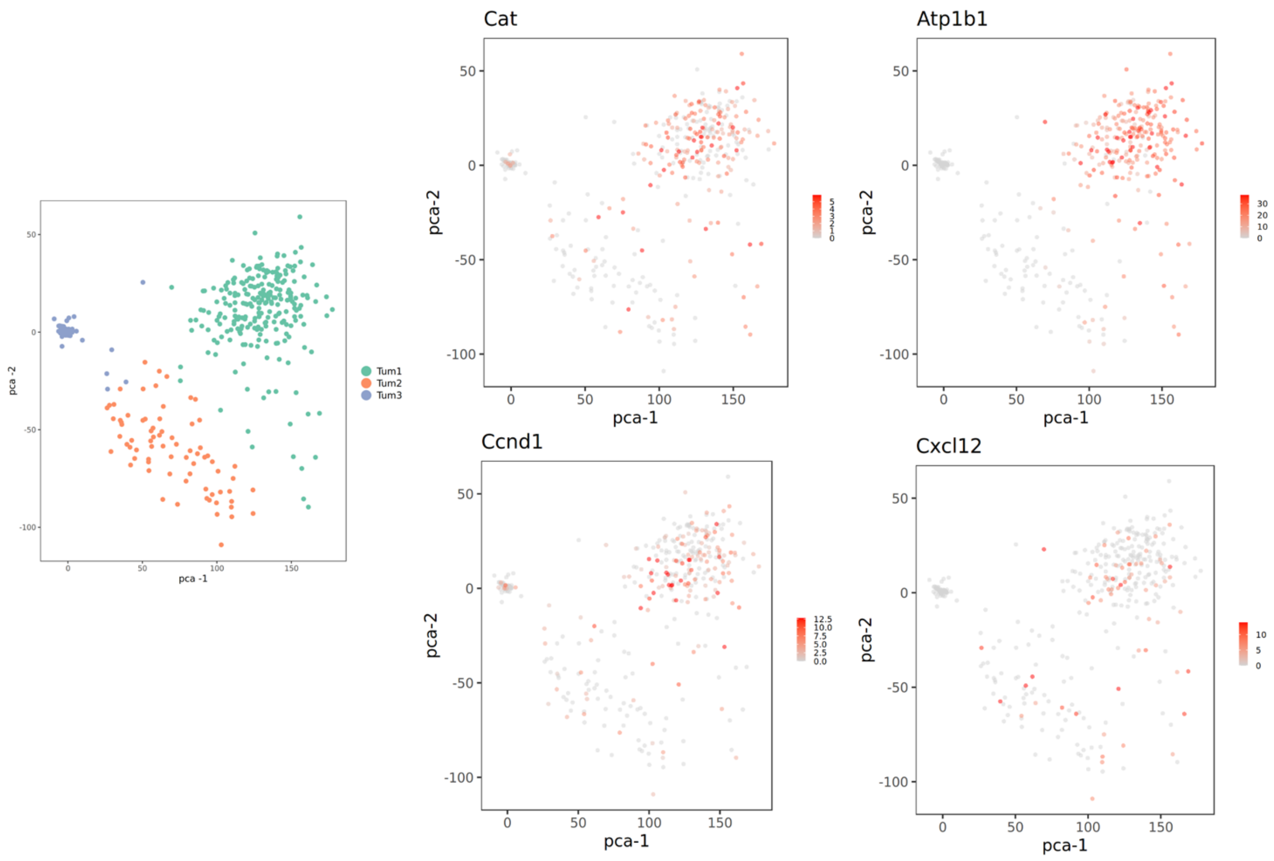

Supplemental Figure 5. PCA plot of the malignant cells colored by the expression level of indicated genes, Cxcl12, Cat, Atp1b1, and Ccnd1.

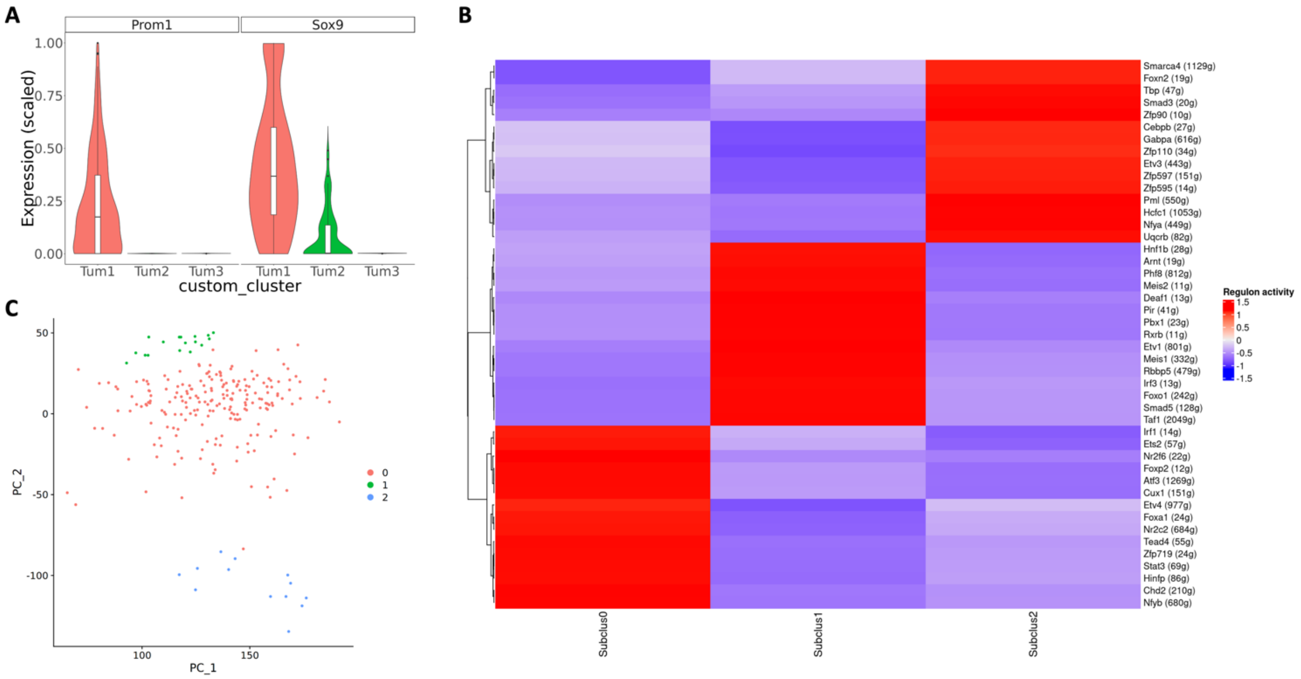

Supplemental Figure 6. (A) A bar-plot showed relative expression level of Prom1 and Sox9 between subclusters of malignant cells. (B) PCA demonstrated the separation of subclusters 0, 1 and 3 in Tum1 cluster colored by sub-clusters. (C) A heatmap of regulon scores from SCENIC analysis. Rows, Individual regulons. Columns, cells organized according to re-clustering of Tum1.

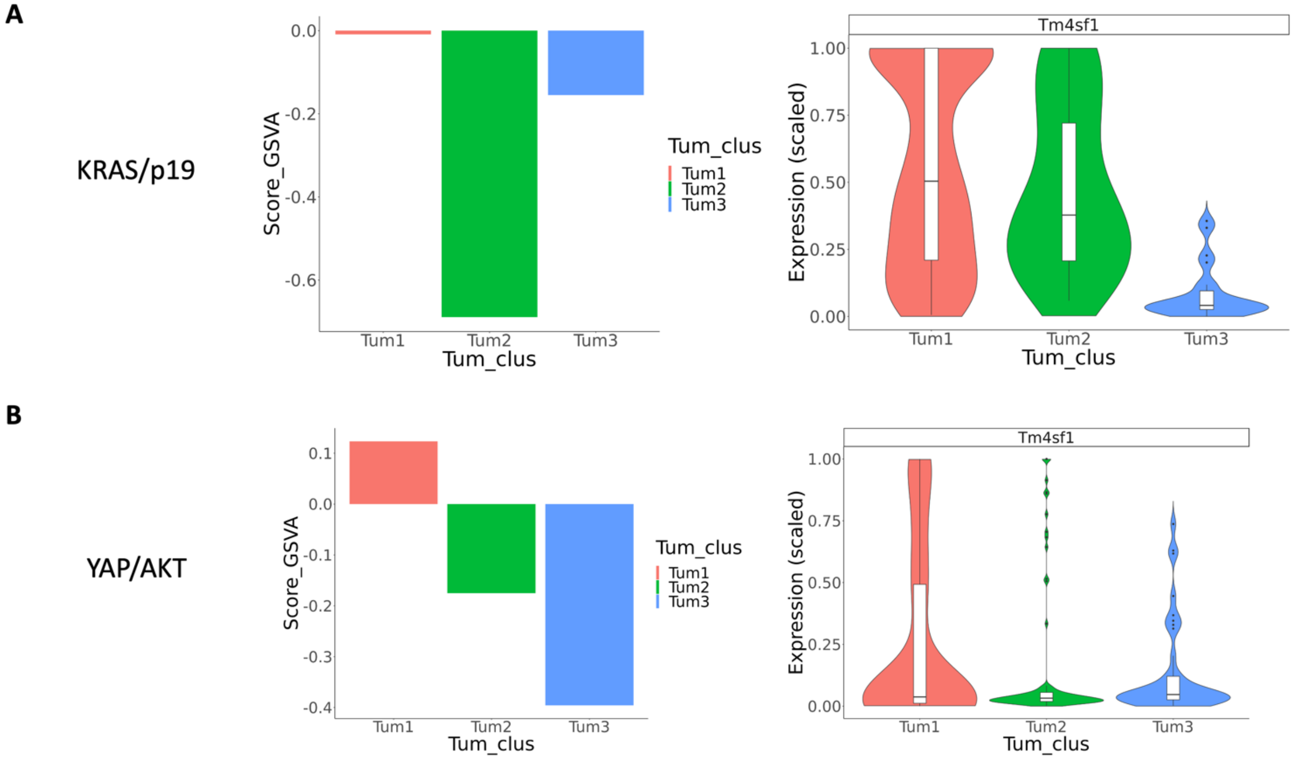

Supplemental Figure 7. (A) Left: A bar-plot showed the average GSVA stemness score for each malignant cell cluster of KRA/p19 CCA mouse model. Right: A bar-plot showed relative Tm4sf1 expression level between subclusters of malignant cells of KRA/p19 CCA mouse model. (B) Left: A bar-plot showed the average GSVA stemness score for each malignant cell cluster of YAP/AKT CCA mouse model. Right: A bar-plot showed relative Tm4sf1 expression level between subclusters of malignant cells of YAP/AKT CCA mouse model.

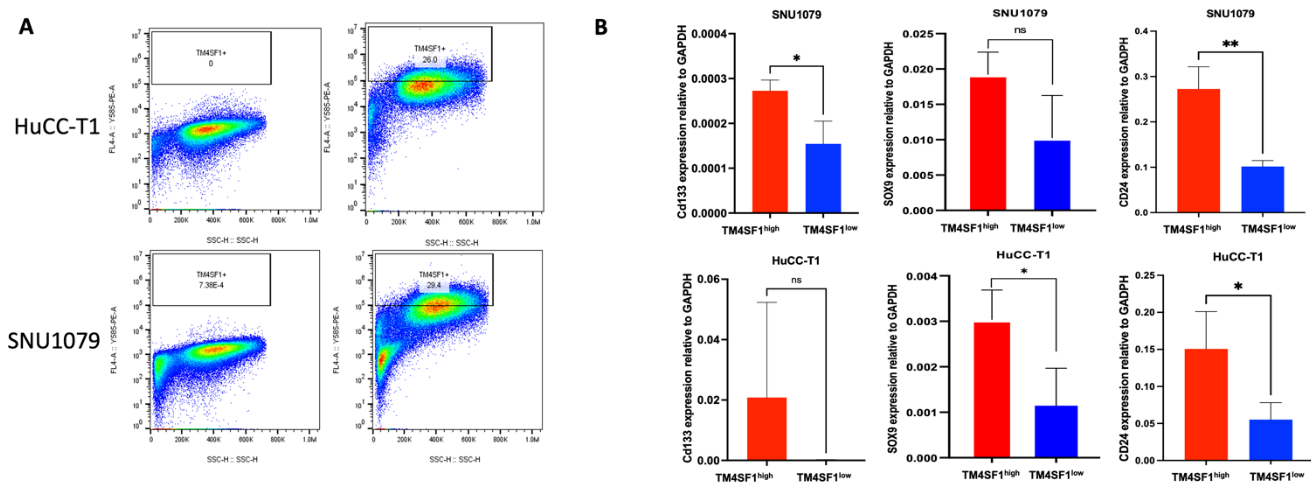

Supplemental Figure 8. (A) Representative of flowcytometry indicated TM4SF1 positivity between different human CCA cell lines. (B) Bar graph showed qPCR analysis of the mRNA expression levels of CSC markers including PROM1(CD133), CD24 and SOX9 in TM4SF1^{high} and TM4SF1^{low} HuCC-T1 and SNU1079 CCA cell lines (3 independent experiments).

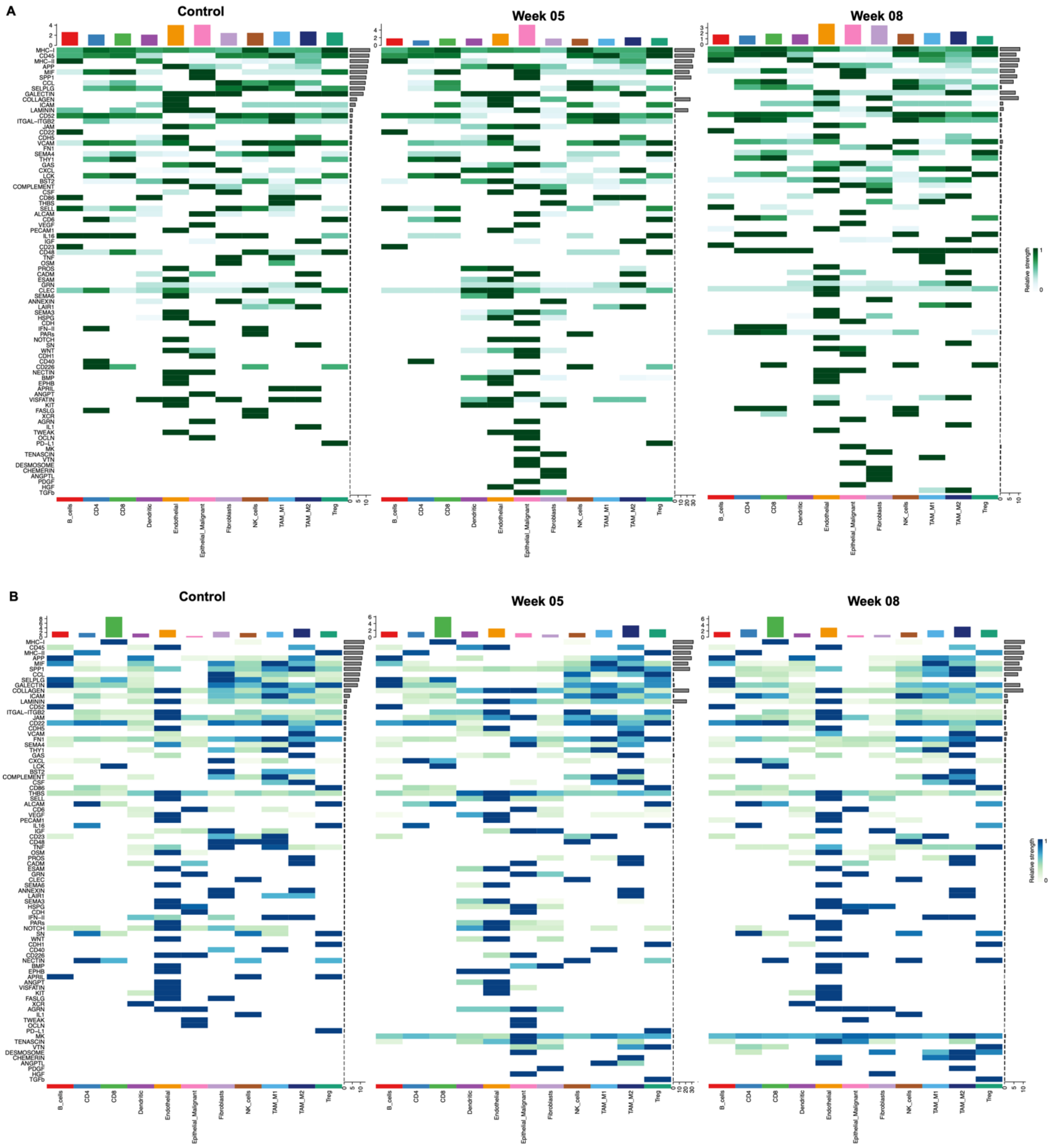

Supplemental Figure 9. Outgoing (A) and incoming (B) signal patterns of malignant cells, cholangiocytes and stromal cells during tumorigenesis of CCA in YAP/AKT mice model. Ctrl, control; W05, week 5; W08, week 8.
