# Supplementary material for: Single-cell RNA sequencing reveals cancer stem-like cells and dynamics in tumor microenvironment during cholangiocarcinoma progression
Source: Front Cell Dev Biol. 2023 Nov 10;11:1250215. doi: 10.3389/fcell.2023.1250215 (PMC10667919; doi:10.3389/fcell.2023.1250215)
Supplement: Supplementary file 6 [file Table5.DOCX]

<https://github.com/NIDAP-Community/Dynamic-of-cancer-stem-like-cells-during-cholangiocarcinoma-progression-/tree/main/Data/Supplementary>
